# Estrogen treatment predisposes to severe and persistent vaginal candidiasis in diabetic mice

**DOI:** 10.1186/2251-6581-13-15

**Published:** 2014-01-08

**Authors:** Mawieh Hamad

**Affiliations:** 1 Department of Medical Laboratory Sciences, College of Health Sciences, University of Sharjah, PO Box 27272, Sharjah, UAE

**Keywords:** *Candida albicans*, CD152, Diabetes mellitus, Estrogen, Immunosuppression, Vaginal candidiasis

## Abstract

**Background:**

Increased levels of estrogen and diabetes mellitus separately predispose to vaginal candidiasis (VC). However, the compounding effect of estrogen on the severity and persistence of VC in diabetic females is not clear.

**Methods:**

To address this issue, a diabetic mouse model with estrogen-maintained VC was developed and evaluated for vaginal fungal burden (VFB) and immune competence at different time points throughout the study period.

**Results:**

Blood glucose levels in estrogen-treated diabetic mice were consistently lower than that in untreated counterparts. Estrogen-treated *C. albicans*-infected non-diabetic mice experienced persistent episodes of VC as compared with naïve controls (*P < 0.01*). However, severity and persistence of VC in estrogen-treated *C. albicans*-infected diabetic mice was significantly greater than that in non-diabetic counterparts (*P < 0.05*). Mortality rates among estrogen-treated *C. albicans*-infected diabetic mice were significantly higher (*P < 0.05*) than that in non-diabetic counterparts. Statistically significant (*P < 0.05*) and persistent suppression of the delayed hypersensitivity response (DTH) was evident in estrogen-treated *C. albicans*-infected diabetic and non-diabetic mice as compared with controls. Levels of expression of the inhibitory molecule CD152 on vaginal and splenic T cells isolated from estrogen-treated *C. albicans* infected mice was significantly higher than that in naive untreated controls (*P < 0.01*).

**Conclusions:**

These findings suggest that estrogen treatment in diabetic females may protect against the progression of DM on the one hand and predispose to severe and persistent VC on the other. The later outcome could be related to the immunosuppressed status of the host.

## Introduction

Vaginal candidiasis (VC) represents a serious health problem to women of childbearing age worldwide [1,2]. Among the significant predisposing factors to VC are increased levels of estrogen in the reproductive tract milieu, diabetes mellitus (DM), and compromised immunity [3-8]. Estrogen acts on both the fungus and the reproductive tract epithelium of the host to enhance fungal adhesion, hyphal growth, and colonization [2,3]. The immunosuppressive effect of estrogen is also thought to play a significant role in the pathogenesis of VC [9,10]. The indispensable role of estrogen in the induction and maintenance of VC is evidenced by the fact that estrogen-dependent VC animal models are routinely used to study various aspects of the pathogenesis and immunity of VC in mice and rats [11-15]. With regard to DM, mounting epidemiological evidence suggest that diabetic females are at greater risk of developing VC than non-diabetic counterparts [11,12,14-16] due to glucose abundance [5] and weakened immunity [16] among other possibilities. Weakened or compromised immunity resulting from the pathogenesis or management of various disease states (cancer, AIDS, organ-transplantation) has also been shown to associate with increased risk of VC [10,17,18].

Increased levels of estrogen in the reproductive tract milieu derive from both endogenous and exogenous sources. Hormonal replacement therapy (HRT) as a source of exogenous estrogen is of particular interest in the context of this study. HRT is commonly used to counter the adverse consequences associating with postmenopausal decrease in estrogen secretion. Such conditions include osteoporosis, neurodegeneration, coronary heart disease, and DM [19-23]. On the other hand, increased risk of breast cancer [24,25] and cardiovascular complications [19] have been linked to HRT. Furthermore, several studies have suggested that postmenopausal women on HRT are at increased risk of VC and RVVC [23,26-28].

Since DM is one of the predisposing factors to VC, it is hypothesized that estrogen treatment in diabetic postmenopausal women could further increase the risk for or enhance the persistence of VC in diabetic hosts, an issue that is yet to be formally addressed. In that, whether the degree of severity and/or level persistence of VC in diabetic females on estrogen therapy is more pronounced than that in non-diabetic counterparts is not known. To address this question, a diabetic mouse model with estrogen-maintained VC was developed and quantitatively evaluated for vaginal fungal burden at different time points of the experiment. Delayed type hypersensitivity (DTH) responses were also evaluated as means of measuring the overall immunocompetence [14,17] of the mouse model described here.

## Materials and methods

### Mice and microorganisms

8-12 weeks old female Balb/c mice raised at the Hashemite University vivarium under non-germ free conditions were used throughout the study. Animal handling and use was in accordance with institutionally-drafted guidelines. A total of 7 mouse groups were prepared each consisting of 68-73 mice. *C. albicans* ATCC strain 36082 used in this study was kindly provided by Dr M. A. Ghannoum (Mycology Reference Laboratory, University Hospital of Cleveland, OH, USA). The fungus was maintained on Sabouraud’s Dextrose Agar (SDA) (Difco, Detroit, MI, USA), stored at 4°C and sub-cultured at 3 month intervals.

### Construction of the animal model

Alloxan-treated mice received a single intraperitoneal (IP) dose of 8% alloxan (65 mg/kg) in 100 μl volumes (Sigma-Aldrich, St. Louis, MO, USA). One-two weeks subsequent to alloxan treatment, estrogen was administered subcutaneously by injecting 0.5 mg of estradiol valerate (Schering AG, Berlin, Germany) dissolved in 0.1 ml sesame oil 72 h prior to *C. albicans* inoculation and at weekly intervals thereafter. Overnight cultures of *C. albicans* were grown at 37°C in SDA broth. Immediately before use, cells were harvested and washed twice in sterile physiological saline (SPS). The vaginal *C. albicans* inoculate consisted of 50μl containing 10^7^ viable stationary-phase blastoconidia. Day of inoculation of mice with *C. albicans* (day zero) marked the start of the experiment. Seven control and experimental mouse groups (Table 1) were evaluated for vaginal fungal burden and serum glucose levels at 13 different time points throughout the study period. At each time point, 5-6 animals/group were tested as appropriate. Select groups were also evaluated for delayed type hypersensitivity (DTH) responses and/or levels of expression of CD152 on vaginal and splenic T cells at specified time points.

**Table 1:** Experimental and control groups included in the study

**Manipulation**	**Mouse group**						
	*1*	*2*	*3*	*4*	*5*	*6*	*7*
Treatment with alloxan	No	No	Yes	Yes	No	No	Yes
Treatment with estrogen	No	No	No	No	Yes	Yes	Yes
Inoculation with *C. albicans*	No	Yes	No	Yes	No	Yes	Yes

### Measurement of blood glucose levels

Plasma glucose measurements were determined by the glucose oxidase method using a Beckman Glucose Analyzer 2 (Beckman Instruments, Fullerton, CA, USA). At each time point, plasma was collected separately from 5-6 mice, plasma glucose concentration was determined and averages were calculated and presented as mean ± SD of three separate experiments.

### Evaluation of vaginal fungal burden

To test for *C. albicans* colonization, 5-6 mice were sacrificed at days 0, 1, 3, 5, 10, 14, 21, 28, 35, 42, 49, 56 and 63 post inoculation; vaginal tissues were isolated, examined for the presence of white lesions characteristic of *C. albicans* infection, trimmed, pooled and homogenized in 10 ml SPS in a sterile glass homogenizer (Ystral GmbH, Gottingen, Germany). Serial 10-fold dilutions were prepared from the homogenate; 1 ml aliquots of the appropriate dilution were added into culture plates containing 10 ml SDA and chloramphenicol at 50 mg/L, plates were left to solidify and then incubated at 37°C; each sample dilution was cultured in triplicate. Yeast colonies were counted 48 hrs after plating and colonization results were expressed as the mean colony-forming unit (CFU) per mouse. Data shown represent the mean ± SD of three separate experiments.

### Measurement of delayed type hypersensitivity

DTH responses were tested as previously described [15]. For each group, separate sets of mice (three-four/group) were right footpad-challenged with 10^7^ heat-killed *C. albicans* in 50μl pyrogen free sterile phosphate saline (SPS) at 2, 3, 4, 5 and 6 weeks post inoculation; left footpad received 50μl SPS as a control. Thickness of the right and left footpads was measured 48 hrs later using a Schnelltaster caliper (H.T. Kroplin Hessen, Schluchtern, Germany). The reaction was counted as positive when the difference in thickness between right vs. left footpad was >0.2 mm.

### Flow cytometric analysis

10^6^ viable cells prepared from vaginal or splenic cell extracts were reacted in 100 μl PBS volumes with FITC-labeled rat anti-mouse CTLA-4 (CD152) (clone 63828) antibody (R&D systems, Emeryville, CA) [29]. Reaction tubes were kept on ice for 20-25 min before fixation (1 ml of 2% Paraformaldehyde/tube). Flow cytometric (FCM) analysis was performed on a Partec PAS flow cytometr (Partec, Münster, Germany) using Flowmax software (Partec) for data acquisition and analysis. Gating of the target population was performed based on lymphocyte physical properties and percentage expression of CD3. Cursors were set based on pre-runs of cell samples stained with isotype-matched control antibodies (Serotec Ltd, Oxford, UK). 50,000 events were collected per sample and percentage positive staining was computed to the 99% confidence level at a logarithmic scale of three decades.

### Statistical analysis

F-test was used to determine levels of significance among different mouse groups with regard to blood glucose levels and vaginal fungal burden (VFB). Student t-test was used to determine the presence of significant differences or lack of it thereof in mortality rates and DTH responses among the various mouse groups.

## Results

**Figure 1:**
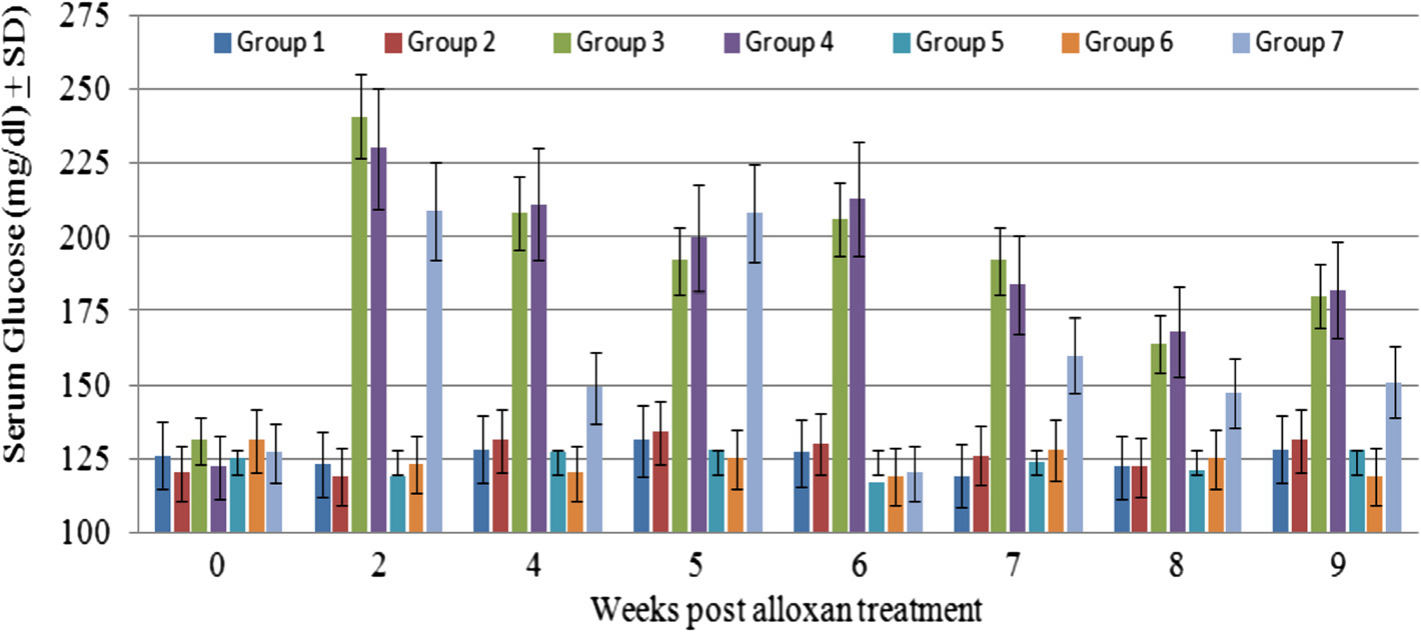
**Serum glucose concentration in control and experimental mouse groups at different time points during the experiment.** Data presented here is the mean serum glucose in mg/dl ± SD of three separate experiments.

As shown in Figure 1, mouse groups treated with alloxan (groups 3, 4, and 7) were persistently hyperglycemic throughout the study period as evidenced by the finding that levels of glucose in these mouse groups were significantly higher than that in untreated mice (*P < 0.01*). Blood glucose levels in mouse groups not treated with alloxan (groups 2, 5, and 6) were similar to that in negative controls (group 1) (*P = 0.2*). No significant differences in blood glucose levels (*P = 0.959*) were observed between non-infected and *C. albicans*-infected diabetic mice. Although glucose levels in group 7 mice were significantly higher than that in mice not treated with alloxan (groups 1, 2, 5, and 6) (*P < 0.01*), it was consistently lower (*P = 0.473*) than that in groups 3 and 4 especially at week 6 onward.

**Figure 2:**
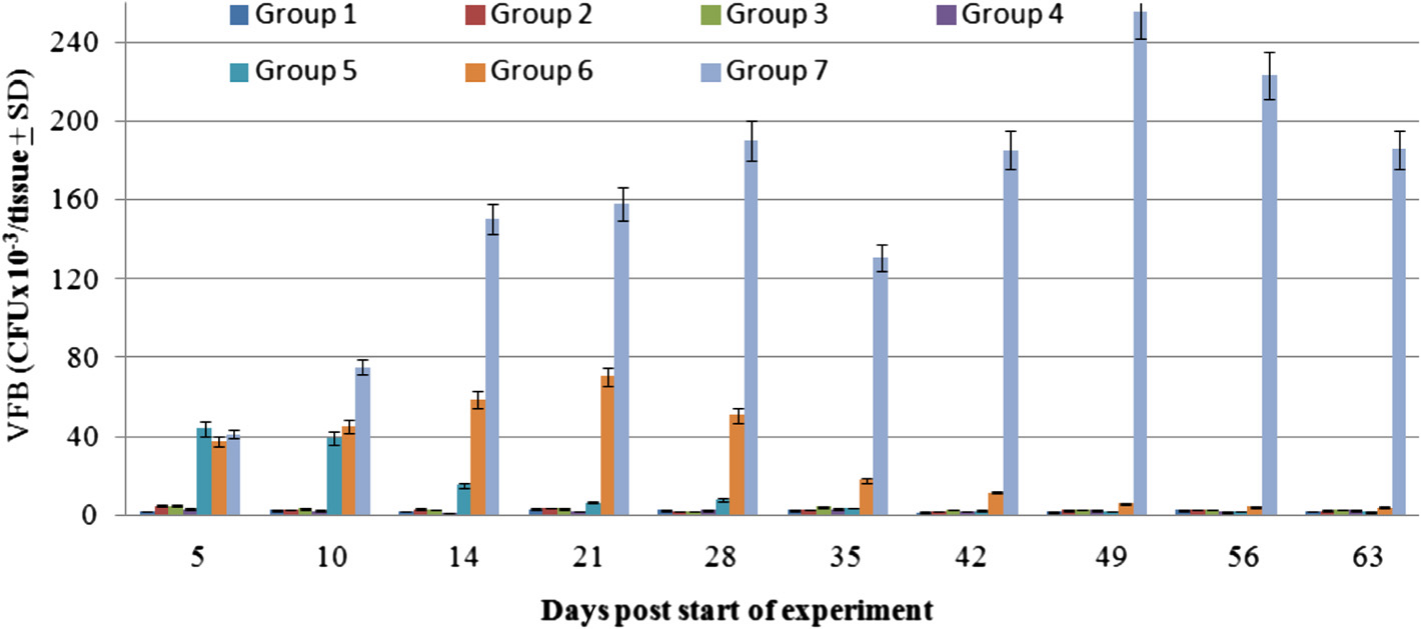
**Vaginal fungal burden in control and experimental mouse groups at different time points during the experiment.** Data represented is the average vaginal fungal burdenx10^-3^/mouse ± SD as obtained from three separate experiments.

Consistent with previous studies [11-16], estrogen treatment resulted in persistent VC for up to 3 and 6 weeks in naïve mice (group 5) and *C. albicans*-infected (group 6) respectively (Figure 2). In both groups, VFB was significantly higher (*P < 0.01*) than that in mice not receiving estrogen treatment (groups 1 and 2). VFB in group 1 was statistically similar to that in naïve diabetics (group 3) (*P = 0.258*) and infected diabetics (group 4) (*P = 0.52*0). Inoculation of estrogen-treated diabetic mice with *C. albicans* (group 7) resulted in persistent and severe VC. VFB in this group was significantly and persistently higher than that in group 1 (*P < 0.01*), group 5 (*P < 0.05*), and group 6 (*P < 0.05*) mice. Unlike the pattern of VC in mice from groups 5 or 6 which tapered off precipitously towards the end of weeks 3 and 6 respectively, group 7 mice experienced very acute episodes of VC that persisted throughout the study period, which lasted for 9 weeks.

**Figure 3:**
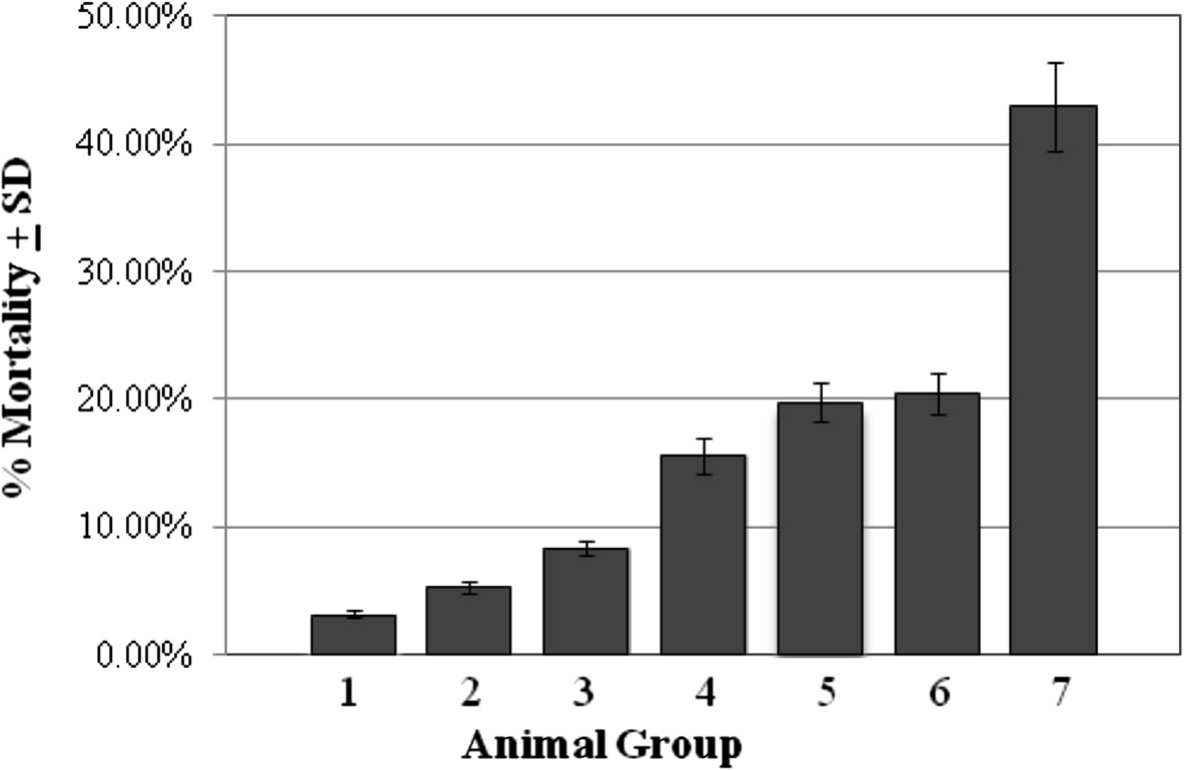
**Mortality rates in control and experimental mouse groups of the study.** Data presented here represent the average percentage mortality/group as calculated by dividing the number of dead mice per total number of mice per group as obtained from three separate experiments.

Rates of mortality in the various control and experimental groups were calculated in order to assess the overall health status of hosts under different conditions. As shown in Figure 3, *C. albicans* infection in the absence of estrogen treatment or diabetes did not result in significant rates of mortality (groups 1 and 2). However, in mice treated with estrogen or alloxan in the presence or absence of *C. albicans* infection, mortality rates were significantly higher (*P < 0.05*) than that in group 1 mice [15]. Noticeably high mortality rates (43%) were observed in group 7 mice, especially at week 5 and afterwards, which were significantly higher than that in any other group (*P < 0.05*). It is worth noting that, starting from week 5 onward, the bladder in the majority of group 7 mice was enlarged, full of urine and whitish in color perhaps signifying severe candidiasis as evidenced by high bladder *C. albicans* CFU counts in comparison with that in naive mice where it was nil (data not shown). Similar qualitative and semi-quantitative observations were made in group 6 mice, albeit at lower frequency and lesser severity.

**Figure 4:**
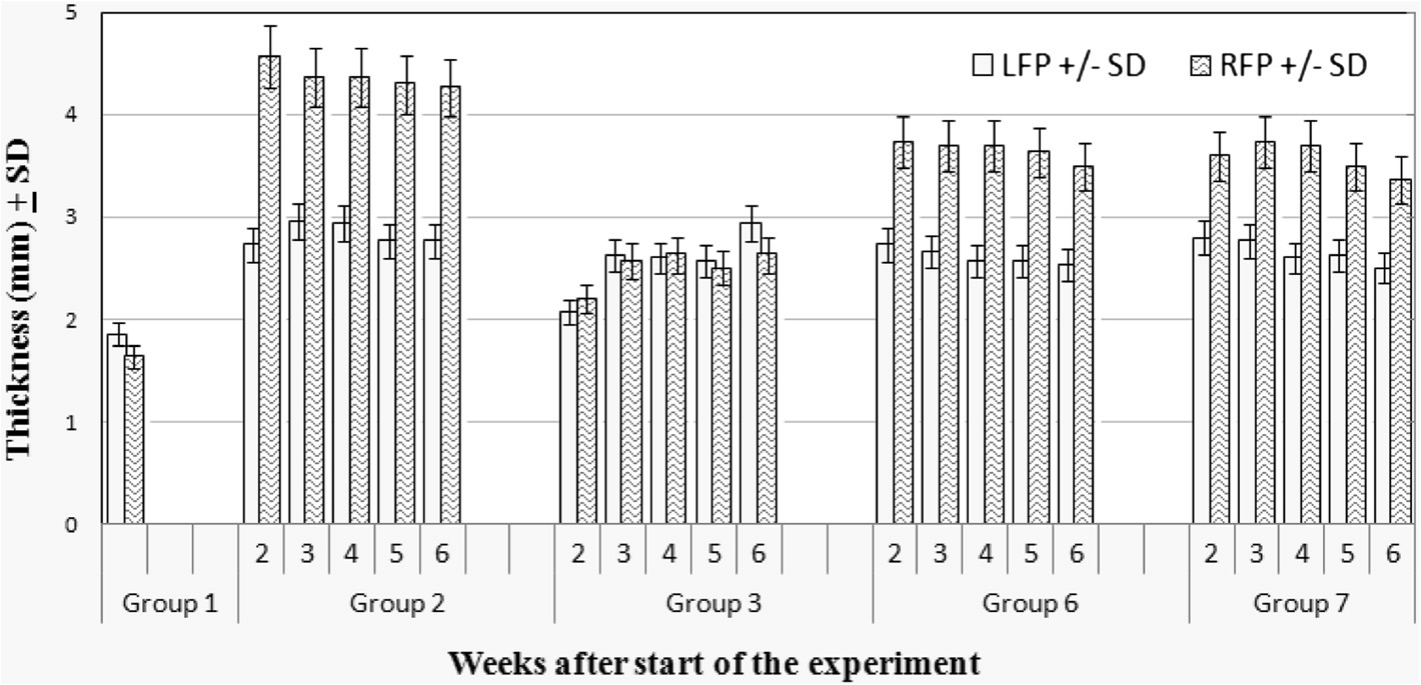
**DTH responses in diabetic and non-diabetic mice in the presence or absence of estrogen and/or *C. albicans *infection.** DTH responses were evaluated by measuring footpad thickness (swelling) in mm 48 hrs after right footpad (RFP) challenge with 50 μl SPS containing 10^7^ heat-killed *C. albicans* cells and left footpad (LFP) with 50 μl SPS. Data represent average footpad thickness ± SD of three separate experiments.

DTH responses against *C. albicans* were evaluated as means of assessing the immune competence of diabetic and/or estrogen-treated mice. *C. albicans*–specific DTH responses were detected in all *C. albicans*-infected mouse groups (groups 2, 6, and 7) as manifested by a >0.2 mm difference between right and left footpad swelling following challenge with heat-killed *C. albicans* blastoconidia (Figure 4). No detectable DTH responses were seen in group 1 or group 3 mice at any time point during the experiment. In agreement with previously published data [14], strong DTH responses were detected in group 2 mice. Such responses, which peaked (4.5 mm) at week 2 post-infection, were significantly higher (*P < 0.01*) than that in mice from group 1 and group 3. Although positive DTH responses were detected in group 6 mice, they were significantly lower (*P < 0.05*) than those observed in group 2 mice. DTH responses detected in group 7 mice were also significantly lower (*P < 0.05*) than that in group 2 mice but statistically comparable to those in group 6 mice (*P = 0.06*).

**Figure 5:**
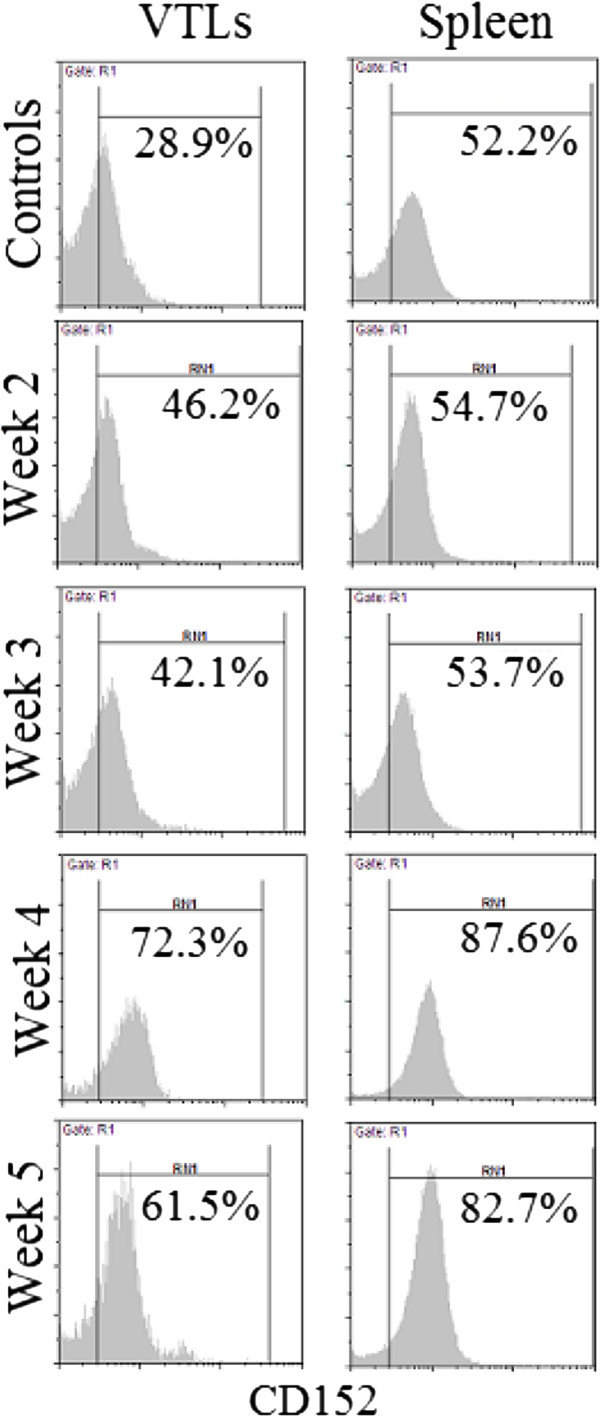
**Levels of expression of CD152 on vaginal (VTLs) and splenic T cells isolated from naïve untreated controls (top panel) and estrogen-treated *C. albicans*-infected mice at weeks 2, 3, 4 and 5 post infection.** Data presented here is representative of three separate experiments.

The effects of estrogen on the immune status of the host was further investigated by periodically measuring the level of expression of the T cell inhibitory molecule CD152 (CTLA-4) on splenic and vaginal lymphocytes isolated from estrogen-treated *C. albicans*-infected mice. As shown in the Figure 5, levels of expression of CD152 were consistently and significantly higher in estrogen-treated *C. albicans*-infected mice as compared with naïve untreated controls (*P < 0.01*). Furthermore, levels of expression CD152 progressively upregulated in the presence of estrogen especially during the first 4 weeks of treatment. In that, an increase of more than 60% in levels of CD152 expression on vaginal and splenic T cells between weeks 2 and 4 was consistently noted in 3 separate experiments.

## Discussion

The experimental model described in this study was helpful in shedding some light on the relationship between estrogen treatment and VC in diabetic females. Consistent with published reports [11-15], estrogen treatment was able to maintain persistent VC in *C. albicans*-infected and naïve mice (Figure 2). Furthermore, estrogen treatment in mice not inoculated with *C. albicans* (Figure 2, group 5) also resulted in relatively mild episodes of VC that persisted for 2-3 weeks clearly suggesting that *C. albicans* is a commensal of the reproductive tract [12,14,15].

More importantly though was the finding that estrogen treatment in *C. albicans*-infected diabetic mice resulted in persistent and protracted episodes of acute VC (Figure 2, group 7). This suggests that estrogen treatment exacerbates VC episodes in diabetic females. This is further supported by the finding that mortality rates (Figure 3) among estrogen-treated *C. albicans*-infected diabetics is significantly higher than that in estrogen-treated *C. albicans*-infected mice (group 6) or in *C. albicans*-infected diabetic mice (group 4).

The finding that estrogen treatment consistently resulted in subdued DTH responses (groups 5, 6 and 7; Figure 4) and upregulated expression of CD152 (Figure 5) may explain how estrogen predisposes to VC. In that, as DTH is a general measure of immune competence [17], subdued DTH responses could be reflective of a state of suppressed immunity, which may in turn enhance microbial pathogenesis. Furthermore, enhanced expression of CD152 on both vaginal and splenic T cells suggests that estrogen treatment leads to localized as well as systemic suppression of T cell immunity; a key player in the defense against VC. These findings are consistent with previous reports which have shown that estrogen treatment associates with weakened DTH responses [14], and upregulated expression of T cell inhibitory molecules like CTLA-4 (CD152) [29]. Estrogen treatment was also shown to induce the expansion of CD4^+^CD25^+^ Treg cells and to enhance their expression of Foxp3 and IL-10 enabling them to suppress naïve T cell proliferation [30] and hence suppress Th1-mediated protective fungal immunity. Estrogen-induced suppression of Th1-mediated protective fungal immunity was also reported to associate with reduced recovery of peritoneal antigen presenting cells, inhibition of inflammatory cytokine (IL-12 and IFN-γ) production, increased production of IL-10 [31], and suppressed production of IL-6 [32].

Several reports have suggested that DM is a contributing factor to VC [1,2,5-8,33]. However, findings reported here suggest that DM on its own neither induces nor maintains VC. This is evidenced by the finding that VFB in naïve diabetics (group 3) and *C. albicans*-infected diabetics (group 4) was comparable to that in naïve mice (group 1; Figure 2). There is the possibility though that differences resulting from the pathogenesis and/or management of DM in humans and animal models not involving the use of alloxan [6,34] may explain this inconsistency. Previous reports have suggested that estrogen treatment could protect against DM [22,33] through, for example, regenerating pancreatic islet β cells and altering the pattern of expression of insulin and progesterone receptors [23]. It was therefore anticipated that estrogen treatment could reverse alloxan-induced hyperglycemia especially in light of previous work which has shown that alloxan-mediated destruction of pancreatic islet β cells involves the activation of anti-islet β cell effector T cells [35,36]. In agreement with these studies [35,36], our findings suggest that estrogen can partially protect against DM as evidenced by the finding that blood glucose levels in estrogen-treated diabetic mice with VC (group 7) were consistently lower than that in untreated diabetic mice (groups 3 and 4) (Figure 1). This is in line with previous studies which have shown that estrogen could exert mild anti-diabetogenic effects (Figure 1) through modulating lipid metabolism [37-39] and hepatic function [40]. Fluctuations in blood glucose levels in group 7, being higher in the early phase (weeks 1-5) of the experiment than the later phase (week 6 onward) is also consistent with the idea that estrogen exerts a protective effect against DM.

In conclusion, findings presented here suggest that although estrogen treatment in diabetic females could partially protect against the progression of DM, it tends to lead to sever and persistent episodes of VC. This should be taken into account in situations involving long-term estrogen treatment; HRT in postmenopausal women is a case in point.

## Competing interests

The author declares that no conflict of interest exists regarding any material described in this manuscript.
